# A nerve-wracking buzz: lessons from *Drosophila* models of peripheral neuropathy and axon degeneration

**DOI:** 10.3389/fnagi.2023.1166146

**Published:** 2023-08-08

**Authors:** Martha R. C. Bhattacharya

**Affiliations:** Department of Neuroscience, BIO5 Institute, University of Arizona, Tucson, AZ, United States

**Keywords:** *Drosophila*, peripheral neuropathy, axon degeneration, ALS, Charcot-Marie Tooth disease

## Abstract

The degeneration of axons and their terminals occurs following traumatic, toxic, or genetically-induced insults. Common molecular mechanisms unite these disparate triggers to execute a conserved nerve degeneration cascade. In this review, we will discuss how models of peripheral nerve injury and neuropathy in *Drosophila* have led the way in advancing molecular understanding of axon degeneration and nerve injury pathways. Both neuron-intrinsic as well as glial responses to injury will be highlighted. Finally, we will offer perspective on what additional questions should be answered to advance these discoveries toward clinical interventions for patients with neuropathy.

## Introduction

Peripheral nerve damage and subsequent degeneration of axons occurs in a diverse group of syndromes and, taken together, makes peripheral neuropathy one of the most frequent human neurodegenerative conditions (Singer et al., 2012). Peripheral neuropathy and peripheral nerve injury occur due to localized injuries like sciatica and carpal tunnel syndrome and via neurotoxicity due to high glucose levels or exposure to microtubule-disrupting chemotherapeutic agents (Theiss and Meller, 2000; LaPointe et al., 2013; Menorca et al., 2013; Albers and Pop-Busui, 2014; Fukuda et al., 2017). In addition, spontaneous or hereditary forms of peripheral neuropathy (including both sensory and motor impairment) can disconnect or disrupt function of circuits (Züchner et al., 2004; Timmerman et al., 2013). Finally, neuroinflammatory diseases like multiple sclerosis cause demyelination and degeneration of axons (Singh et al., 2017). Because of the relative ease of access to peripheral structures compared to those in the central nervous system, peripheral nerves are an attractive setting for studies of degenerative pathways that also affect the brain.

Pathological axon degeneration is also called Wallerian degeneration due to its initial description in the frog hypoglossal nerve by Waller (1851). Even Waller (1851) recorded macroscopic findings that match what we see today: a delay period following injury, a rapid axon fragmentation process, and the subsequent clearance of the fragmented nerve. We now have more sophisticated tools to watch this process both *in vitro* and *in vivo* in many organisms (Tao and Rolls, 2011; Shin et al., 2012; Babetto et al., 2013; Vargas et al., 2015; Tian et al., 2016; Li et al., 2018). The molecular study of axon degeneration was launched by a serendipitous discovery of the Wallerian Degeneration Slow (Wld^s^) mouse phenotype and, later, the responsible genomic alteration [triplication of the NAD-producing enzyme, nicotinamide mononucleotide adenylyltransferase (NMNAT1)] (Lunn et al., 1989; Coleman et al., 1998; Mack et al., 2001). These discoveries showed that axon degeneration, rather than a passive process, was actively controlled: the initiation, pace, and extent are determined by the activation state of cellular pathways. This led to a flurry of research that has identified the key components of the Wallerian degeneration pathway (summarized below) (Miller et al., 2009; Bhattacharya et al., 2012, 2016; Osterloh et al., 2012; Shin et al., 2012; Xiong et al., 2012; Neukomm et al., 2017) and led to emerging strategies for clinical intervention in peripheral neuropathy (DiAntonio, 2019; Feldman et al., 2022; Shi et al., 2022).

*Drosophila melanogaster* has led the way in identifying the molecular pathways downstream of axon injury that result in axonal fragmentation and disassembly. Using fly models of genetic, traumatic, or toxic nerve injury, mutations in multiple fly genes including *Drosophila* sterile alpha and TIR motif containing protein (*dSarm*), *highwire* (*hiw*), and *wallenda* (*wnd*) genes were first identified as axo-protective (Miller et al., 2009; Osterloh et al., 2012; Xiong et al., 2012). Following initial descriptions of pathways in fly, later work in mouse has universally confirmed the key *Drosophila* discoveries (Table 1). Given this excellent foundation, fly models of peripheral neuropathy and axon degeneration continue to be a key source of information about the mechanisms activated by nerve injury and strategies to mitigate its effects.

**TABLE 1:** Genes discovered in *Drosophila* for roles in axon degeneration.

Gene (fly)	Gene (mouse)	Function in axon degeneration	Molecular role	Cell type	Fly discovery references	Mammalian references
*sarm*	*SARM1*	Pro-degenerative	NAD^+^ enzymatic destruction	Neurons	Osterloh et al., 2012	Osterloh et al., 2012
*highwire (hiw)*	*PHR1*	Multiple roles	E3 ubiquitin ligase	Neurons	Xiong et al., 2012	Babetto et al., 2013
*wallenda (wnd)*	*DLK1*	Pro-degenerative	MAP3K	Neurons	Miller et al., 2009	Miller et al., 2009
*skpA*	*SKP1A*	Pro-degenerative	Component of E3 ubiquitin ligase	Neurons	Brace et al., 2014	Yamagishi and Tessier-Lavigne, 2016
*axundead (axed)*	TBD	Pro-degenerative	Ubiquitin tagging of proteins	Neurons	Neukomm et al., 2017	N/A
*retinophilin (rtp)*	*MORN4*	Pro-degenerative	Membrane tethering of myosin	Neurons	Bhattacharya et al., 2012	Bhattacharya et al., 2012; Mecklenburg et al., 2015
*draper (drpr)*	*MEGF10*	Debris clearance	Signaling to activate MMP-1 and phagocytosis	Glia	MacDonald et al., 2006; Logan et al., 2012	Wu et al., 2009
*tmep*	*TMEM184B*	Pro-degenerative	Endolysosomal control	Neurons	Bhattacharya et al., 2016	Bhattacharya et al., 2016
*wnk*	*WNK1*	Axo-protective	Serine-threonine kinase	Neurons	Izadifar et al., 2021	Izadifar et al., 2021
*raw*	TBD	Pro-degenerative	Restrains JNK signaling	Neurons	Hao et al., 2019	N/A
nmnat*	*NMNAT1,2,3*	Axo-protective	NAD^+^ production	Neurons	Fang et al., 2012; Xiong et al., 2012	Mack et al., 2001

Shown are fly gene names and abbreviations, mouse gene names, functions in axon degeneration, molecular roles, and cell types in which each protein acts. References provided are for the initial discovery of roles in axon degeneration in *Drosophila* or mouse. In the mouse reference column, N/A indicates that a clear mammalian ortholog of the fly gene has not been identified. An asterisk indicates that NMNAT was first identified as axo-protective in mice and later confirmed to be conserved in fly Wallerian degeneration.

**FIGURE 1:**
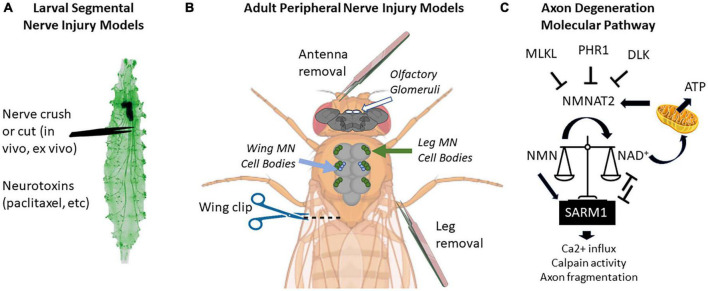
Locations of peripheral nerve injuries commonly used in *Drosophila* melanogaster and molecular pathway of axon degeneration. **(A)** Larval segmental nerves (green) can be crushed with forceps or cut with scissors. Larvae can also be fed drugs that cause nervous system toxicity, such as paclitaxel. **(B)** Peripherally projecting axons of central nervous system structures (brain and thoracic ganglion) can be severed by removing appendages such as antennas or legs or by cutting through the wing margin. On the brain, the location of olfactory glomeruli is shown with white circles. On the thoracic ganglion, the location of cell bodies of motor neurons (MN) from the legs is shown in green, while the cell bodies from wing neurons are shown in blue. **(C)** Current proposed pathway of axon degeneration centered around regulation of NMNAT2 and SARM. Gene names use mammalian nomenclature. A high NMN/NAD^+^ ratio triggers Sarm1 activation, which triggers a positive feedback loop to further reduce NAD^+^ via enzymatic cleavage. NMNAT2 is the predominant axonal form in mammals, but axonally localized NMNAT1 can provide a similar function. Not pictured: Axed, Wnk1, or intermediate MAP kinase pathway members [MKK4/MKK7 and c-Jun n-terminal kinase (JNK)]. Larval image modified from Balapagos and used under Creative Commons ShareAlike Genetic license (CC BY-SA 2.0). Image creation was assisted by Biorender.com.

Larval and adult stage *Drosophila* have many accessible peripheral nerves from which to choose for neuropathy studies (Figure 1). In larvae, the body is organized in repeating hemi-segments, and nerves innervate each hemi-segment in a stereotyped pattern. The larval nerves contain both sensory and motor axons, as in mice. Because these nerves leave the VNC in close proximity to each other, a researcher can injure 6–8 nerves simultaneously in one animal (or leave them as uninjured controls) (Brace and DiAntonio, 2020). In adults, nerves entering appendages such as antennae, legs, and wings are ideal models for neuropathy studies due to their accessibility (Phelps et al., 2021). Two other advantages of adult axon degeneration models (versus larval studies) are (1) they allow for a longer analysis of axon injury effects (up to 60 days), and (2) the function of these axons can be read out behaviorally using olfactory, locomotor, or grooming assays (Wang et al., 2011; Neukomm et al., 2017; François-Moutal et al., 2019; Paglione et al., 2020).

In this review, I will highlight new findings from *Drosophila* that have potential to offer both better mechanistic understanding of different types of neuropathy and relevant directions for future patient care. The main (though not exclusive) focus will be on peripheral nerve injuries and disease models in *Drosophila*, though axon degeneration certainly occurs in central nervous system disorders such as Alzheimer’s Disease and tauopathies (Law et al., 2022). I will also offer some caveats of the *Drosophila* models that should be considered when extrapolating findings to mice and man.

## Injury and toxicity-induced Wallerian degeneration in peripherally projecting nerves

Physical crush or transection of fly nerves is perhaps the most utilized neuropathy-inducing insult because it permits synchronization of the injury time course in multiple axons and also because the injury is localized to a single site for each nerve. Using olfactory antennal axotomy (by removal of one of the two antennae, which retains viability), early candidate approaches and unbiased screens identified Wallerian degeneration factors such as dSarm1 and Wallenda (Miller et al., 2009; Osterloh et al., 2012). In a parallel screening paradigm using transection of wing axons, the axonal localization of mitochondria was found to be critical for axonal maintenance following injury (Fang et al., 2012). Together, these three screens set the stage for a large expansion of this field and, importantly, established *Drosophila* as a strong gene discovery platform for evolutionarily conserved nerve injury factors (Miller et al., 2009; Sasaki et al., 2009; Bhattacharya et al., 2012, 2016; Xiong et al., 2012; Babetto et al., 2013; Neukomm et al., 2014).

Sarm1 (the mammalian ortholog of *dSarm) is* widely appreciated to be the “central executioner” for axon degeneration (Osterloh et al., 2012; DiAntonio, 2019). Initially characterized as an adaptor for Toll-like receptors (Couillault et al., 2004; Liberati et al., 2004; Carty et al., 2006), Sarm1 was unexpectedly found to have enzymatic activity that cleaves NAD^+^ molecules, reducing their availability in neurons and causing metabolic catastrophe (Essuman et al., 2017). It is this NAD-destroying function that precipitates the rapid degeneration of axons *in vitro* and *in vivo* (Geisler et al., 2019; Bosanac et al., 2021). To probe the domains responsible for this action, multiple groups have built transgenic *Drosophila* lines that express structurally altered variants of dSarm or Sarm1 (Neukomm et al., 2017; Brace et al., 2022; Herrmann et al., 2022). These lines enabled the discovery of separate signaling functions of Sarm1 in developmental versus degenerative signaling, highlighting multiple ways that this NAD-cleaving enzyme contributes to nervous system integrity (Brace et al., 2022; Herrmann et al., 2022).

The pathway for activation of Sarm1 is still under investigation, but clues have recently emerged. In one study by Izadifar et al. (2021), With-No-Lysine (K) (Wnk) kinase was discovered to be an inhibitor of Sarm1 activity in both *Drosophila* and mice. Loss of dWnk causes axon degeneration in multiple model systems, and its over-expression prevents some Sarm1-mediated disruption of neural circuits (Izadifar et al., 2021). Recently it was also found that nicotinamide mononucleotide (NMN), a synthetic precursor of NAD^+^, binds to an allosteric site on the armadillo (ARM) repeat domain and promotes Sarm1 activation. As the NMN/NAD^+^ ratio increases, Sarm1 activity also increases (Figley et al., 2021). It will be of future interest to examine how Wnk kinase expression affects the metabolic state of the neuron, which could link these two observations.

Intersecting with the dSarm/Sarm1 pathway, the proteins Highwire [PAM-Highwire-Rpm-1 (Phr1) in mammals] and Wallenda [dual leucine zipper kinase 1 (Dlk1) in mammals] also promote injury-induced degeneration and were first recognized for this role using injuries in the fly larval segmental nerves and the olfactory nerves, respectively (Miller et al., 2009; Xiong et al., 2012). Work in both fly and mouse systems demonstrated that Hiw/Phr1, an E3 ubiquitin ligase, targets an axonally-transported NMNAT variant, NMNAT2, for degradation (Gilley and Coleman, 2010; Xiong et al., 2012; Babetto et al., 2013). Thus, when Hiw/Phr1 is absent, NAD^+^ is produced in higher levels in the axon, providing axo-protection after injury. The E3 ubiquitin ligase complex containing Highwire also downregulates a pro-degenerative pathway controlled by the MAP triple kinase (MAP3K) Wallenda/DLK (Collins et al., 2006). Downstream of Wallenda/DLK and other MAP3Ks, multiple factors ensure the fragmentation of injured or NAD^+^ -depleted axons (Shin et al., 2012; Huntwork-Rodriguez et al., 2013; Larhammar et al., 2017).

Axon degeneration in *Drosophila* can also be initiated by treatment with drugs that induce nerve damage. For example, in humans, microtubule-disrupting components of conventional chemotherapy cocktails disrupt axon transport in long axons, leading to their demise. Using the drug paclitaxel (taxol) to trigger peripheral axon degeneration in *Drosophila* larvae, additional neuronal axon degeneration factors were identified including membrane occupation and recognition nexus 4 (MORN4) and transmembrane protein 184b (TMEM184B) (Bhattacharya et al., 2012, 2016). How these factors fit into known signaling pathways, specifically those controlled by Sarm and by MAP kinase cascades, remains to be determined.

Recent studies have expanded the focus from neuron-intrinsic factors to reveal information on the role of glia in injury responses. Flies have glia that cover the range of functions expected of mammalian glial types, including those for nerve insulation (wrapping glia), barrier function (subperineurial and perineurial glia), synaptic regulation (astrocyte-like glia) and neuronal cell body contacts (cortex glia) (Logan and Freeman, 2007; Yildirim et al., 2019). *Drosophila* subperineurial glia also have engulfment functions in both development and disease (Sonnenfeld and Jacobs, 1995). In the absence of injury, peripheral axons in *Drosophila* must be maintained via appropriate wrapping and metabolic signaling; these processes are controlled by the discoidin domain receptor (Ddr) and TGFβ signaling, respectively (Corty et al., 2022; Lassetter et al., 2023).

Following axonal injury, the *Drosophila* glial surface receptor Draper initiates the phagocytic pathway involved in severed axon clearance (MacDonald et al., 2006). Using appendage axotomy to robustly activate glial injury responses, the evolutionarily conserved signaling pathway downstream of Draper was recently identified (Lu et al., 2017; Purice et al., 2017). Draper signaling initiates transcriptional changes in glia following nerve injury (Lu et al., 2017; Purice et al., 2017). This cascade proceeds via transcriptional activation by STAT92E and AP-1. Through this transcriptional regulation, matrix metalloproteinase MMP-1 gene expression is upregulated and is necessary for remodeling of tissues and glial membrane expansion following injury (Purice et al., 2017). Other differentially activated glial genes have yet to be fully characterized in this system, but overall the analysis points to both Draper and Toll receptor signaling as key determinants of phagocytic and immune responses to injury.

## Inherited sensory neuropathy models

*Drosophila* models of Charcot-Marie-Tooth (CMT) syndrome have yielded important insights into the pathological mechanisms causing sensory and motor neuropathy. CMT is linked to mutations in over 100 genes (WU Neuromuscular, 2023) and can be classified into myelin-affecting (CMT1, CMT4) and axon-affecting (CMT2, CMTX) phenotypes (Bolino and D’Antonio, 2023). Regardless of the subtype, all CMTs ultimately result in axon degeneration of peripheral nerves. *Drosophila* models of CMT2-associated mutations in Mitofusin2 (MFN2), have revealed that both loss- and gain-of-function human variants of MFN2 cause CMT-associated neurodegeneration and have implicated excessive mitochondrial fusion and disrupted mitochondria-ER contacts in disease pathogenesis (El Fissi et al., 2018; Shen et al., 2021). In another CMT subtype (CMT4J), autosomal recessive mutations in the PIP_2_ phosphatase FIG4 in humans cause a syndrome showing degeneration both in central and peripheral neuron populations (Chow et al., 2007). In mice, FIG4 mutation reduces the number of large diameter myelinated axons and impairs nerve conduction and action potential generation (Chow et al., 2007). By altering *Drosophila* Fig4 to introduce mutations corresponding to human patient variants, we now know that impaired phosphatase activity is not the reason for the patient syndrome and that its role in the nervous system involves the maintenance of lysosomal membrane integrity (Bharadwaj et al., 2016). By taking advantage of the “rough eye” phenotype caused by dFig4 mutations in flies, a modifier screen recently resulted in the identification of long non-coding RNAs that whose knockdown can suppress some phenotypes of dFig4 (Muraoka et al., 2018; Shimada et al., 2020). Whether this phenotype is due to a direct effect on Fig4 expression levels or on other related pathways has not yet been determined, but it may offer clues for how to molecularly target the Fig4 pathway in CMT 4J patients.

Spinal muscular atrophy (SMA), which causes motor neuron loss, can be modeled in *Drosophila* by depleting the survival motor neuron (SMN) protein; these models also exhibit motor neuron death. By taking advantage of the extremely well-described trajectory of motor neuron cell fate decisions during *Drosophila* embryonic development (Mark et al., 2021; Meng and Heckscher, 2021), Grice and Liu (2011, 2022) were able to show that early neurogenesis disruption, in addition to later neuromuscular junction (NMJ) dysfunction, contribute to locomotor phenotypes in SMA.

Some limitations to extrapolation of *Drosophila* findings to mammalian systems should be considered. First, *Drosophila* do not myelinate their nerves. In some peripheral neuropathies such as CMT Type 1A, Schwann cell disruption by mutations in peripheral myelin protein 22 (PMP22) or myelin protein zero (MPZ) cause secondary axon degeneration (Bolino and D’Antonio, 2023). The lack of myelination in *Drosophila* suggests this subtype is better modeled in vertebrates. Second, *Drosophila* lack an adaptive immune system. Therefore, non-neuronal responses to nervous system injury are likely simplified when compared to their mammalian counterparts. In the mouse, for example, sequential post-injury infiltration of neutrophils, macrophages and lymphocytes into injured nerves contribute to sensitization and pain (Marchand et al., 2005).

## Dendrite degeneration

Patients with poorly controlled diabetes often experience peripheral neuropathy, which presents as hyperalgesia (extra sensitivity to normally innocuous stimuli), which can later progress to numbness. Inter-epidermal nerve fibers in the skin that subserve these sensations can show dystrophic terminals and withdrawal from the epidermis (Cheung et al., 2015). Patients with painful diabetic neuropathy also show decreases in intraepidermal nerve fiber density, indicating that nerve terminals are disrupted (Cheung et al., 2015). *Drosophila* fed a high sugar diet show thermal nociceptive hypersensitivity, similar to painful neuropathy in humans (Dabbara et al., 2021). This phenomenon is driven by sensory neuron function: when insulin receptor expression is impaired specifically in sensory neurons, overall dendrite length of multidendritic Class IV sensory neurons (which tile the cuticle wall) is reduced and animals show persistent hyperalgesia (Im et al., 2018). Thus the *Drosophila* system is well suited to examine not only pathological axon damage but also dendrite dysfunction.

Recent work has highlighted the similarities and differences between axon and dendrite degeneration by taking advantage of the class IV sensory neurons described above. In this system, Sarm over-expression or NMNAT knockout cause dendrite degeneration, suggesting that dendrites also degenerate via NAD^+^ depletion (Ji et al., 2022). The process of fragmentation was found to be separable from that of phagocytic clearance, done by endothelial cells in the larval cuticle. The authors describe a dendrite degeneration model which proposes three NAD^+^ “checkpoints:” one which activates Sarm, one in which additional Sarm-induced NAD^+^ exposes phosphatidylserine (PS) on the surface of injured dendrites to recruit phagocytic cells, and finally an ultra-low NAD^+^ level that causes dendrite breakdown independent of phagocytosis (Ji et al., 2022). These intriguing results suggest that there are more levels of control of neurite breakdown than previously appreciated. Further work will be required to confirm the alterations in NAD^+^ levels directly and to examine whether this model extends to mechanisms in axons or in other locations where glia rather than epidermal cells are responsible for phagocytosis.

## Motor neuropathy and ALS models

The *Drosophila* larval neuromuscular junction has long been appreciated as a phenomenal location for studies of synaptic function; indeed, significant findings on the genes controlling the synaptic vesicle cycle were initially identified using this platform (Zhang, 2003; Frank et al., 2020). Using flies to model motor neuropathies such as amyotrophic lateral sclerosis (ALS) thus has been very well received. One caveat to the point-by-point comparison to mammalian systems is that, in mammals, the neuromuscular junction is cholinergic rather than glutamatergic. However, basic principles of neural toxicity of ALS-linked proteins are easily modeled in *Drosophila* larval and adult peripheral nerves (Yang et al., 2015). Some studies also use photoreceptor neurons or the central brain to investigate neuron death associated with ALS-linked gene expression (Ihara et al., 2013; Cunningham et al., 2020; Dubey et al., 2022). Here we will focus on axonal and synaptic effects of ALS genes in *Drosophila*.

The simplicity of *Drosophila* transgenic creation has permitted many models of ALS in which human or *Drosophila* versions of disease-associated protein variants can be expressed in spatially and temporally specific ways using binary expression systems like GAL4-UAS (Brand and Perrimon, 1993). For example, TDP-43, a protein whose aggregation is a hallmark of ALS and other neurodegenerative diseases, can be expressed in *Drosophila* larval motor neurons or in accessible adult structures (eyes, legs, or wings) to evaluate the pathways leading to motor neuron dysfunction and loss (Li et al., 2010; Estes et al., 2011; Sreedharan et al., 2015). Using pupal lethality as a readout of TDP-43 toxicity, an innovative drug screen for ALS suppressors identified pioglitazone, a PPARγ agonist, as a compound that mitigates TDP-43-dependent locomotor dysfunction (Joardar et al., 2015). However, this compound does not protect against all genetic causes of ALS and its mechanism is still unclear. Metabolomic profiling of larvae with TDP-43 over-expression has also revealed a role for the Tricarboxylic acid (TCA) cycle in the pathology of ALS (Loganathan et al., 2022).

In 2011, two groups studying ALS cohorts identified a hexanucleotide expansion in chromosome 9, open reading frame 72 (C9orf72) as a cause of ALS, and this alteration has since been appreciated to be the most common genetic alteration in sporadic ALS cases (DeJesus-Hernandez et al., 2011; Renton et al., 2011). While no C9orf72 homolog exists in flies, *Drosophila* models have been instrumental in understanding how this hexanucleotide expansion, which causes expression of multiple dipeptide repeat proteins, cause neurodegeneration. One of the biggest strengths of fly models is the ability to perform *in vivo* forward genetic screens, which have identified mediators and modifiers of dipeptide repeat toxicity (Xu et al., 2013; Solomon et al., 2018; Goodman et al., 2019). *Drosophila* models are also used to validate *in vitro* screening results in cultured cells (Donnelly et al., 2013). For example, stress granule accumulation occurs in ALS-affected neurons and disrupts critical cellular functions like nucleocytoplasmic transport and RNA metabolism (Li et al., 2013; Zhang et al., 2015, 2018). Markmiller et al. (2018) performed a screen in the *Drosophila* eye for suppressors of the ALS-associated “rough eye” phenotype and identified a number of novel candidates. Candidates were also studied directly in peripheral nerves of the *Drosophila* wing, and 5 novel stress granule proteins were identified as modifiers of the ALS phenotype (Markmiller et al., 2018).

## *Drosophila* as a validation platform for newly discovered neuropathy genes

Whole genome sequencing of rare disease patient samples, and genome-wide association studies, are continuously generating predictions of the importance of individual genetic loci to disease risk or severity. In order to demonstrate a true effect of these genetic variants, it is often required to modify the gene’s expression (up or down), either alone or in the background of a disease model. The costs and time associated with doing these rigorous experiments in mice *in vivo* is substantial. In contrast, *Drosophila* is a simple and powerful system in which genetic manipulations are simple and time-efficient, and appropriate disease models (such as those described above) are easily identified. Because it is predicted that roughly 65% of human disease genes have orthologs in *Drosophila* (Ugur et al., 2016), the fly has become a preferred model in which to first test the consequences of altering genes predicted by human genomic analyses (Sarkar and Feany, 2021; Wang et al., 2021).

The use of the fly to test disease-associated variants can be done in multiple ways such as transgenic expression of disease variants, knockdown of fly ortholog(s) of the human genes, or directly modifying the analogous amino acids in the fly ortholog via CRISPR. To give a few examples: Sorbitol dehydrogenase (SORD) homozygous mutations were recently identified in a cohort of patients clinically diagnosed with CMT. By creating a *Drosophila* model in which the orthologs (Sord1 and Sord2) were disrupted, the authors showed not only that the role of SORD in axon maintenance is conserved but that a pharmacological reversal of the phenotype is possible (Cortese et al., 2020). In another example, Cytochrome c oxidase assembly factor 7 (COA7) was identified from patient genomic analysis as a new causative gene for peripheral neuropathy (Higuchi et al., 2018). To model this disorder *in vivo*, the authors created a *Drosophila* model of COA7 impairment and showed defects in synaptic branches of the peripheral terminals of motor neurons as well as impaired locomotion (Higuchi et al., 2018). These results supported the causality of the variant-phenotype relationship *in vivo*.

## Discussion

Considering the advantages in accessibility, cost, and evolutionary conservation described above, *Drosophila* has become appreciated as one of the best models for understanding both neuron-intrinsic and extrinsic contributions to peripheral nerve maintenance and pathological forms of peripheral nerve loss. The number of human genomics studies who complete their first *in vivo* validations in *Drosophila* is growing. Additionally, a network of laboratories has united to use model organisms as rare disease profiling platforms, with Drosophila as one of the leaders in this movement (Gahl et al., 2015; Huang et al., 2022; Morimoto et al., 2023; Srivastava et al., 2023). For more established models of diseases such as ALS, flies will continue to provide a platform for unbiased, *in vivo* screening for enhancers and suppressors of nerve degeneration and cell loss.

A goal for future work on nerve injury in *Drosophila* is to unite our understanding of how glial cells contribute to axonal damage responses. This field is still in its infancy. However, because of the conservation of functions of glia in *Drosophila* and mammals, glial-neuronal communication can be addressed well in flies using advanced genetic tools and forward screening approaches (Yildirim et al., 2019). Another challenge that lays ahead is to integrate the pathways identified through human disease cohorts into known signaling pathways mediating axon degeneration (for example, are processes disrupted by new neuropathy genes like SORD or COA7 upstream or downstream of Sarm1?). An exciting era is before us as we begin to answer these questions.

As mentioned above, caveats do exist for this model involving lack of myelination or an adaptive immune system. However, the myriad advantages of using *Drosophila* to identify and characterize factors contributing to axon, dendrite, and synapse disruption will continue to keep flies at the leading edge of discoveries that, in turn, will improve our ability to mitigate the suffering of patients suffering from peripheral neuropathy.

## Author contributions

MB performed the research, wrote the manuscript, and approved the submitted version.
